# Osseous Metaplasia in an Inflammatory Polyp of the Rectum: A Case Report and Review of the Literature

**DOI:** 10.4021/gr417w

**Published:** 2012-03-20

**Authors:** Brian R. Odum, Matthew L. Bechtold, Alberto Diaz-Arias

**Affiliations:** a Department of Pathology and Anatomical Sciences, University of Missouri-Columbia, USA; b Department of Internal Medicine, University of Missouri-Columbia, USA

**Keywords:** Osseous metaplasia, Colon polyps, Inflammatory polyps

## Abstract

Osseous metaplasia is a rare finding in colonic neoplasms. We report a case osseous metaplasia in a 74 year-old male who underwent surveillance colonoscopy and found to have a 7 mm rectal polyp. Histopathologic examination revealed an inflammatory polyp with osseous metaplasia.

## Introduction

Osseous metaplasia is a phenomenon described in a wide variety of tissue types in relation to both neoplastic and non-neoplastic conditions. In the setting of neoplasia it is seen in both benign and malignant lesions. The mechanism responsible for this metaplastic change is not completely understood, and its impetus remains unidentified. Furthermore, osseous metaplasia is exceedingly rare in colon polyps. Individual cases have been reported, often with accompanying literature reviews, describing the phenomenon and theories regarding potential pathogenesis. In these literature reviews, it seems that several cases are often overlooked, most citing 5 - 10 cases total. We report a case of osseous metaplasia in an inflammatory rectal polyp with an accompanying literature review, summary of seventeen previously reported cases of osseous metaplasia in a variety of colon polyps, and a novel proposed mechanism for its pathogenesis.

## Case Report

**Figure 1:**
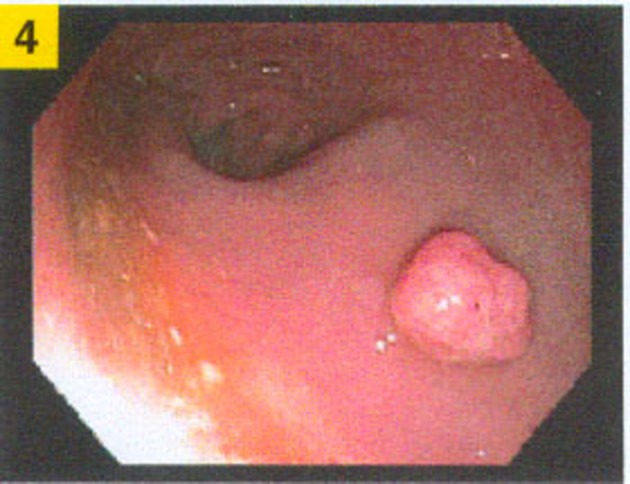
Rectal polyp during colonoscopy.

A 74 year-old male with history of prior tubular adenoma of the ascending colon removed via snare polypectomy, hyperlipidemia, coronary artery disease status post angioplasty with stent placement, prior traumatic left frontotemporal subdural hematoma status post evacuation, urethral stricture status post multiple dilatations with recurrent urinary tract infections, and an anucleate right eye secondary to childhood trauma presented for a surveillance colonoscopy. At colonoscopy, a 7 mm rectal polyp was discovered which was removed by cold snare (Fig.1).

**Figure 2:**
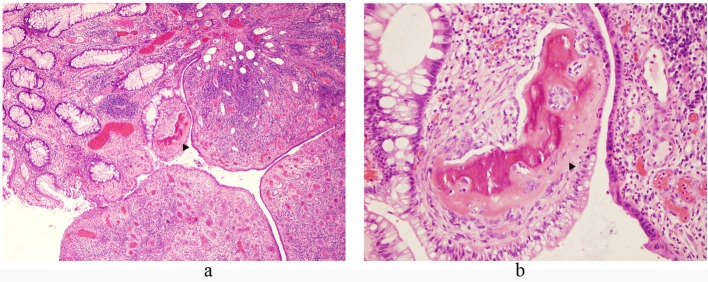
Microscopic images of H&E stained sections of the inflammatory rectal polyp with focus of osseous metaplasia (arrows); (a) 40 x magnification, (b) 200 x magnification.

On pathologic gross examination, the polyp was a 1.0 x 0.4 x 0.4 cm fragment of tan-red mucosal tissue that was bisected and submitted for histologic examination. Microscopically, H&E stained sections of the polyp showed fragments of denuded colonic mucosa with an edematous and congested lamina propria, overlying fibrinopurulent debris, and adjacent granulation tissue (Fig. 2). Within the intact colonic mucosa, the crypts were elongated and branching with dilatation and abundant mucin production. No dysplasia was identified. The granulation tissue consisted of a fibrous stroma with abundant prominent small blood vessels and a marked mixed acute and chronic inflammatory infiltrate. At the junction of the intact colonic crypts and the adjacent granulation tissue, there was a small focus of woven bone formation. No bone marrow was present within the bone fragment.

## Discussion

Osseous metaplasia is rarely encountered in colon polyps. Seventeen cases have been reported in the reviewed literature (Table 1).Of the previous cases, eight have been reported in polyps with low grade dysplasia; specifically three in tubular adenomas [1-3], four in tubulovillous adenomas [4-8], and one in a serrated adenoma [9]. One of the tubular adenomas occurred in the sigmoid colon [1], one was found in the transverse colon [2], and one was from an undisclosed site [3]. One of the tubulovillous adenomas was seen in the cecum [4], one was in the sigmoid [5], and one was in the rectum [6, 7]. The location of the fourth tubulovillous adenoma was not disclosed [8]. The serrated adenoma was discovered in the recto-sigmoid portion of the colon [9]. Additionally, nine cases of osseous metaplasia have been reported in benign colon polyps [6, 10-16]; specifically three in inflammatory polyps [10-12] and six in juvenile polyps [6, 13-16]. All three of the inflammatory polyps occurred in the rectum [10-12]. Three of the juvenile polyps were reported in the rectum as well [6, 13, 14], while the fourth was from an undisclosed site [15]. The fifth and sixth juvenile polyps were reported in terms of their distance from the anus; one noted at 14 cm (rectosigmoid) and the other at 10 cm (rectum) [16].

**Table 1:** Summary of the Current Case and Previously Reported Cases of Osseous Metaplasia in Colon Polyps Including Corresponding Reference

Polyp Type	Location	Size (mm)	Age (yrs)	Gender	Year	Reference
Dysplastic						
Tubular Adenomas	Sigmoid	15	85	F	2005	(1)
	Transverse	Unknown	63	F	2008	(2)
	Unknown	12	59	M	1997	(3)
Tubulovillous Adenomas	Cecum	20	73	M	1999	(4)
	Sigmoid	25	Unknown	Unknown	2000	(5)
	Rectum	18	67	M	1994	(6, 7)
	Unknown	Unknown	Unknown	Unknown	1996	(8)
Serrated Adenomas	Rectosigmoid	25	50	M	2010	(9)
Benign						
Inflammatory Polyps	Rectum	12	39	M	2009	(10)
	Rectum	10	25	M	1981	(11)
	Rectum	10	22	F	1992	(12)
Juvenile Polyps	Rectum	Unknown	10	M	1964	(13)
	Rectum	20	3	F	1994	(6)
	Rectum	10	15	M	2009	(14)
	Unknown	Unknown	Unknown	Unknown	1963	(15)
	Rectosigmoid	10	5	M	1992	(16)
	Rectum	5	4	M	1992	(16)
Current Case Inflammatory Polyp	Rectum	10	74	M	2011	

Osseous metaplasia is a well-described phenomenon, but it is extremely rare. A review of cases at an academic medical center over a 10 year period revealed 85 cases of heterotopic ossification out of 125,000 surgical pathology specimens. Twenty-two of the cases were neoplastic lesions, and the remaining 73 were non-neoplastic [17]. Osseous metaplasia is well described in the setting of intestinal neoplasia, both benign and malignant [17-19]. The mechanism of osseous metaplasia is not well understood. Theories regarding potential mechanisms have evolved considerably since the earliest case reports. A set of experiments published by Huggins in 1931 demonstrated osseous metaplasia in abdominal wall soft tissue after transplantation of bladder tissue with intact urothelium to abdominal wall fascia in dogs. These experiments provided evidence that some component of the epithelial elements may prompt the ossification of mesenchymal tissue [20]. In 1939, Dukes published a case series of osseous metaplasia in rectal carcinomas suggesting that common features of the phenomenon were long duration of symptoms indicating a slow growing tumor, the histologic picture of a low grade tumor with little potential for metastasis, and the presence of areas of necrosis within the tumor [18]. Van Patter and Whittick, in 1955, published a series of cases of osseous metaplasia in intestinal neoplasms and suggested that the phenomenon resulted from the interaction of local physicochemical factors, like calcium salts and mucin, with proliferating connective tissue within certain tumors [19]. In a 1963 report of osseous metaplasia in a juvenile polyp, Todd interpreted the finding to support the view that juvenile polyps are malformations rather than neoplasms [15]; a view that is readily discounted by the occurrence of osseous metaplasia in both dysplastic and frankly malignant lesions. In 1964, Marks and Atkinson suggested that osseous metaplasia may result from the ability of fibroblasts to transform into other types of mesodermal tissue, specifically osteoblasts [13]. That theory is the basis for a majority of modern investigations into the origin of osseous metaplasia.

Current evidence points toward the differentiation of mesenchymal cells into osteoblasts, which subsequently produce bone. Immunohistochemical (IHC) analysis of tissue with osseous metaplasia, by Randall et al in 1989, demonstrated alkaline phosphatase expression, a marker of synthesis, in the osteoblast-like cells of metaplastic bone and to a lesser degree in the glandular cells of metastatic colonic adenocarcinoma and adjacent proliferating mesenchymal cells [21]. More recent studies have focused on the expression of bone morphogenetic proteins (BMPs) in the setting of osseous metaplasia [17, 22, 23]. Most BMPs are members of the TGFβ superfamily and play an integral role in the formation of new bone, except BMP-1 which is a metalloprotease and a marker for cartilaginous differentiation. Detection of BMP-2, BMP-4, BMP-5, and BMP-6 by IHC in colonic adenorcacinomas with osseous metaplasia was demonstrated by Imai et al in 2001. BMP-2, BMP-4, BMP-5 and BMP-6 were present in the cytoplasm of tumor cells and within the osteoblast-like cells of newly formed bone, and BMP-2 and BMP-4 were noted within stromal fibroblasts [22]. Kypson et al, in 2003, demonstrated the overexpression of BMP-2 in tumor cells from cases of rectal adenocarcinoma with osseous metpalasia, compared to cases of rectal adenocarcinoma lacking bone formation. They suggest this lends credence to the theory that some the epithelial cells provide the stimulus for heterotopic bone formation [23]. In 2007, Liu and colleagues demonstrated the variable expression of BMP-1, BMP-4, and BMP-6 by IHC in the stroma and epithlium related to osseous metaplasia in a variety of settings [17]. Taking a different approach, Chauveau and colleagues, in 2004, demonstrated increased expression of osteocalcin and upregulation of type-1 collagen and osteonectin, all markers of bone matrix synthesis, in metaplastic bone through analysis of mRNA by PCR [24]. Clearly the exact mechanism of osseous metaplasia is still a topic of significant debate.

Though the *in vivo* mechanics of osseous metaplasia are still under investigation, the initial step down the path of osteoblastic differentiation is still unknown. Perhaps shedding some light on the subject, in *in vitro* studies, mouse fibroblasts cultures under the influence of a four specific transcription factors (Oct3/4, Sox2, c-Myc, and Klf4) have generated pluripotent stem cells. Subsequently these stem cells have demonstrated the capability to differentiate into cell types from all three germ cell layers [25, 26]. Additional study of adult human fibroblasts, *in vitro*, has demonstrated a similar phenomenon [27]. Perhaps mature fibroblasts present within the stromal component of these intestinal lesions are under the influence of these or other similar transcription factors, leading to the generation of stem cells capable of differentiating into osteoblasts. Evidence to support this theory is limited, and additional research is warranted.
